# Changing Clinical Presentation, Current Knowledge-Attitude-Practice, and Current Vision Related Quality of Life in Self-Reported Type 2 Diabetes Patients with Retinopathy in Eastern India: The LVPEI Eye and Diabetes Study 

**DOI:** 10.1155/2016/3423814

**Published:** 2016-10-24

**Authors:** Taraprasad Das, Batriti Wallang, Preeti Semwal, Soumyava Basu, Tapas R. Padhi, Mohd Hasnat Ali

**Affiliations:** ^1^LV Prasad Eye Institute, Hyderabad, India; ^2^LV Prasad Eye Institute, Bhubaneswar, India

## Abstract

*Purpose*. To document the changing clinical presentation of diabetic retinopathy (DR) over a decade, the current knowledge-attitude-practice (KAP) of known type 2 diabetes mellitus (DM) patients, and the current vision related quality of life (VR-QOL) of patients with DR in a tertiary eye care center in Eastern India.* Methods*. Two hundred and forty patients with known type-2 DM were evaluated. The evaluation included status of DR (*n* = 240), KAP (*n* = 232), and VR-QOL (*n* = 75). International classification of DR was used in the study. The DR status was compared with another cohort (*n* = 472) examined a decade earlier, in year 2001. The KAP-25 questions were designed after literature review. The National Eye Institute Visual Function Questionnaire (NEI-VFQ; including optional items) was validated by Rasch analysis. Both KAP and VR-QOL were analyzed according to degree of DR, duration of known DM, and educational qualification.* Results*. Average age of the current cohort (*n* = 240) was 57.16 ± 9.03 years; there were 205 (85.4%) male patients and 143 (59.6%) patients had received less than graduate qualification. The mean duration of DM since diagnosis was 10 ± 7.8 months (range 8 months to 30 years); 118 (49.16%) patients had DR. In a decade time, 2001 to 2011, there was a change of retinopathy status at presentation (more often nonproliferative diabetic retinopathy, NPDR). One-third of NPDR patients had poor vision and half of them were hypertensive. KAP was better in patients with higher education and those having DR. VFQ score was higher in better seeing patients.* Conclusion*. Patients currently presenting at earlier stage of retinopathy are probably related to poor vision. Early detection and treatment of DR is likely to preserve and/or improve vision.

## 1. Introduction

Diabetes mellitus (DM) is a condition of growing concern with increasing prevalence worldwide and associated high morbidity [1]. It is projected that 522 million people are likely to have DM by year 2030 in the world (International Diabetes Federation) and this increase is disproportionately more in developing countries (69% versus 20% with 2010 as baseline) [2]. It will result in a heavy burden on the health care system because of several DM related complications. Diabetic retinopathy (DR), an important complication of DM, is the leading cause of visual disability in diabetics. The reported prevalence of DR in India ranges from 17.6% to 28.2% [3–6]. With this prevalence, the number of people with DM is expected to increase from current 62 million to 79.4 million and patients with DR increase to 22.4 million in another two decades [2]. The potential economic and social impact of this increasing burden calls for a definite need for an effective screening strategy, accurate case detection, and treatment effective for both DM and DR. 

DM is related to both genetics and the life style. Awareness of DM and practice to keep it under control depends on the patient and the provider. DR treatment strategy is related to the stage of retinopathy. The status of DR dictates vision related quality of life (VR-QOL). The LV Prasad Eye Institute Eye and Diabetes Study (LEADS) was designed to address some of the common questions related to diabetes care in self-reported type 2 diabetes patients presenting to a tertiary eye care center in the institute's Bhubaneswar (Eastern India) campus. This prospective study addressed the following three issues in patients with DR:

1. The current pattern of diabetic retinopathy at presentation vis-à-vis a decade earlier.
2. The current knowledge-attitude-practice (KAP).
3. The current vision related quality of life (VR-QOL).

## 2. Materials and Methods

**Figure 1:**
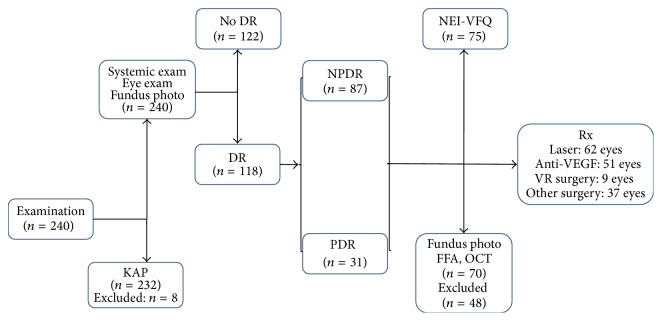
LEADS steps and patients flow.

The LEADS protocol approved by the institutional ethics committee (LEC 11-093) was a prospective study of the eye and systemic condition of patients with DM presenting to the LV Prasad Eye Institute. Consecutive self-reported type 2 diabetes patients reporting to the institute for the entire period of November (month of World Diabetes Day) 2011 were recruited into the study. The overall study protocol involved a systematic 3-step evaluation as illustrated in Figure 1 and adhered to tenants of Helsinki. The 1st step involved a comprehensive eye examination, basic systemic evaluation, and the KAP assessment. Collected data included the demographic details (age, gender, and education), the status of systemic condition (known duration of DM and current treatment, comorbidities, and treatment), and previous ophthalmic intervention. Eye examination included the presenting and best corrected visual acuity (BCVA) for distance recorded by the ETDRS chart placed at 4 meters and near vision at 33 cms, slit lamp examination that documented any significant anterior chamber pathology, Goldmann applanation intraocular pressure, and crystalline lens status. A detailed fundus examination using indirect ophthalmoscope by a trained retina fellow was followed by fundus photograph by Forus fundus camera (Forus, Bangalore, India). The systemic examination involved the anthropometric measurements (height by measuring tap fixed to the wall, weight by a digital weighing machine, abdominal girth by a measuring tap, and body mass index), every time done by the same observer (BW), and the blood tests (HbA1c, serum creatinine, and lipid profile). All patients detected clinically to have DR were classified as either nonproliferative diabetic retinopathy (NPDR) or proliferative diabetic retinopathy (PDR) using the International Diabetic Retinopathy classification and entered the 2nd step of evaluation. Patients with no clinical DR were advised to return for a follow-up examination or were treated for the other eye condition as required.

The 2nd step involved a repeat fundus photograph by Zeiss fundus camera (Carl Zeiss FF 450 Plus IR, Dublin, USA), fundus fluorescein angiography (FFA; Carl Zeiss FF 450 Plus IR, Dublin, USA), and optical coherence tomography (OCT; Carl Zeiss, Dublin, USA). The NEI-VFQ questionnaire (for international comparison) was administered to all patients with DR. The patients who needed further treatment for DR entered into the 3rd step of care (laser or intravitreal anti-VEGF or vitreous surgery).

*Diabetic Retinopathy at Presentation*. We compared our 2011 data with the ones we had collected a decade ago in November 2001 (unpublished) for a similar group of self-reported type 2 DM patients. We compared the DR status (any DR and no DR), DR type (NPDR and PDR), BCVA, and comorbidities (hypertension and cardiac) (Table 1). The proportion of each category was compared (chi square test of 2 proportions; Medcalc) and *p* < 0.05 was considered significant.

### 2.1. KAP

The KAP questionnaire was developed after a review of the literature on guidelines of earlier KAP studies. Our KAP questionnaire consisted of 25 multiple choice questions on both DM and DR. The divisions of questions were as follows: the knowledge questions (1) to (10); the attitude questions (11) to (18); and the practice questions (19) to (25) (Tables 2 [Table tab3]–4).

A printed copy of the questionnaire in English was given to each enrolled patient within the 1st step of the LEADS after a written consent was obtained. The instructions for completing the questionnaire were provided on the face sheet and were also explained verbally by one of the authors (BW). The non-English speaking or less literate patients were helped in completing the questionnaire by translation from a trained volunteer. The patients were given the option of ticking more than one option for each question should they feel there was more than one correct answer.

The number of patients providing correct response was calculated for each question. The knowledge questions were also individually analyzed for the number of patients knowing each correct option or factor. With regard to the practice questions an answer was regarded as correct if the patient was compliant with the treating physician or ophthalmologist's instructions. A cut-off value less than 55% was taken as “not good.” Subgroup analysis was done for each question according to the duration of DM (<20 years and ≥20 years), education (<graduate and ≥graduate), and DR status (presence and absence of DR). Comparison of KAP was done for each subgroup and analyzed by chi square test (*p* < 0.05) for statistical significance.

### 2.2. VR-QOL

The National Eye Institute 25-item Visual Function Questionnaire (NEI-VFQ 25) [7] plus optional items questionnaire is a supportive survey for determining the problem of vision specific health condition in patients with chronic eye disease. This instrument was used so that it could be compared with similar studies in other countries. The VFQ 25 plus optional items (VFQ 39) are composed of 11 vision targeted conceptual elements with an additional general health scale. The 11 vision targeted constructs are directed for the following: general vision; ocular pain; near activities; distance activities; social functioning; mental health; role difficulties; dependency; driving; color vision; and peripheral vision. As per the guidelines, each of these 12 subscales was given a score ranging from 0 (lowest) to 100 (highest). The total VFQ score was calculated by taking average of all vision related subscales, except the general health subscale. The total score, therefore, ranged from 0 to 100 and higher score indicated better vision specific activities.

VFQ questions were administered to patients clinically diagnosed to have DR. One of the authors (PS), trained to administer such questions, administered the questionnaire after explaining and obtaining a written consent. All instructions written on the cover page were verbally explained, read, and described; the list of answers was read briefly and the patient was given complete freedom to answer.

*Rasch Analysis*. All statistical analyses were performed using R software (version R 2.14.1). Rasch analysis was performed using R package eRM to determine the validity of the NEI-VFQ 25 [8]. Initially we fitted the Rasch model with all 25 items except items 15 and 16 (because of high missing data). We used Masters Partial Credit model using conditional maximum likelihood examination, as there are different types of rating scales. To test the unidimensionality axiom, we performed Martin-Lof test. Item-fit and person-fit statistics were used to detect misfit data in the Rasch model. According to this, items included in subscales of general health, general vision, ocular pain, and driving were removed. After removing the misfit items we refitted the Rasch model. The subscales were analyzed separately using the same procedures.

Group analysis was done based on the disease spectrum (NPDR and PDR) and best corrected visual acuity (≥20/60 and <20/60). Comparison of groups was done using unpaired* t*-test (*p* < 0.05). Multiple regression model with stepwise elimination using Akaike information criteria was used to assess the relationship between the vision related QOL and demography (age, gender, and educational qualification) and clinical variable (presence of comorbidity, BMI, serum creatinine, HbA1c, presence of macular edema, and better eye BCVA). The final model retained the following factors: age, presence of comorbidity, presence of macular edema, and better eye BCVA.

## 3. Results

The LEADS enrolled 240 patients. This included 205 (85.4%) male and 35 (14.6%) female patients; the mean age was 57.16 years ± 9.03 months (range 31–80 years). These recruits were consecutive and surprisingly there were less female patients (no specific reasons). One hundred and forty-three (59.6%) patients had received less than graduate qualification. All patients had type 2 DM. The mean duration of DM since detection was 10 ± 7.8 months (range 8 months to 30 years). DR was detected in 118 (49.16%) patients; 87 (73.7%) had NPDR and 31 (26.7%) had PDR. In patients with DR, 39 (33%) patients had macular edema and 12 (10%) patients had sight threatening retinopathy. Seventy-eight patients with clear media (absence of significant cataract and vitreous hemorrhage) were advised to have FFA and OCT. One or more comorbidities were present in 172 (71.66%) patients and hypertension detected in 126 (52.5%) patients was the commonest comorbidity. Two patients had bilateral poor vision (<20/200).

The 2001 cohort had enrolled 472 self-reported type 2 DM patients. There were 368 males and the average age was 57.7 years ± 10.4 months (range 29–82). The DR status at presentation in 2001 and 2011 and other demographic details are shown in Table 1.

Table 1 lists the major differences in clinical presentation of patients in years 2001 and 2011. We observed 4 significant changes in 2011 patient cohort compared to 2001 patient cohort: (1) more often DM patients consulted ophthalmologist before they developed DR; (2) more often patients presented in NPDR than in PDR state; (3) lower number of patients had good vision (≥20/60); and (4) hypertension was more prevalent. The cohorts of 2001 and 2011 were different and any of the patients of earlier cohort was not included in the later cohort.

### 3.1. KAP

Two hundred thirty-two patients completed KAP questionnaire (8 patients refused). There were 199 males (85.8%) and mean age was 57 ± 8.9 years (range 31–80 years). One hundred forty-three patients (61.6%) were less than graduates.

*Knowledge (Table 2) (Questions (1)–(10))*. General knowledge of DM was reasonably good (questions (1), (2), (4), and (5)) though only two-thirds of patients were aware of necessary life style modification (question (3)). Eye and other organs complications were the commonest known DM complication (question (5)). The overall knowledge of DR was generally poor (questions (7)–(10)) though two-thirds of patients had knowledge that retina is affected in DR (question (6)). In general, there was a trend towards better knowledge in patients with higher education (questions (5) and (8)).

*Attitude (Table 3) (Questions (11)–(18))*. Only two-thirds of patients were aware of the need for life long medication (question (11)). Patients had a good attitude for regular eye check (questions (12) and (16)) though many thought it was required only on experiencing visual symptoms or when advised by treating diabetologist (questions (13), (15), and (18)). Close to half patients thought that blood sugar control is key to prevention of DR (question (14)) and that laser treatment of the eye is a permanent cure of DR (question (17)). Patients with higher education and patients with DR had a comparatively better attitude for eye examination (questions (14) and (17)).

*Practice (Table 4) (Questions (19)–(25))*. Practice regarding regular checkup of blood pressure, eye examination, regular intake of medicines, and maintaining a controlled diet (questions (20)–(22) and (24)) was good. But practice towards foot care and exercise was not good (questions (23) and (25)). In general patients with higher education and patients with DR had a better practice (questions (22) and (23)).

### 3.2. Vision Related QOL

Seventy-eight patients (who were advised for FFA and OCT) were eligible for VR-QOL. Three patients refused to give written consent for the questionnaire; hence NEI-VFQ was administered to 75 patients with DR. This cohort of 75 patients comprised 66 (88%) males; 51 (68%) patients had received less than graduate qualification; mean age was 57.88 ± 7.51 (range 39–77 years) years; mean known DM duration was 14.23 (range 10 months–30 years) years. Better eye BCVA was <20/60 in 37 (49.3%) patients. Fifty (66.7%) patients had one or more comorbidities, predominantly hypertension (43 patients; 86%). Following FFA and OCT, 54 (72%) patients were confirmed to have NPDR, 21 (28%) patients to have PDR, and 39 (52%) patients to have macular edema. The outfit (unweighted) and infit (weighted) mean square statistics, chi square, and* p* value are given in item-fit statistics.

Based on the disease spectrum (Table 5), NPDR, and PDR, there was no significant difference in VFQ score. While near activities, mental health, dependency, color vision, peripheral vision were better in patients with NPDR, social functioning was better in patients with PDR; but none of these factors were statistically significant. Based on BCVA, the VFQ score was higher in patients with better vision (≥20/60) (Table 6). This included near and distance activities, social functioning, mental health, role difficulties and dependency, color, and peripheral vision. Multivariate analyses further confirmed the importance of BCVA affecting the VFQ score. Other important independent factors affecting composite score were increasing age and better vision (better VFQ score) and presence of comorbidity (worse VFQ score). Presence of macular edema showed a trend towards worse VR-QOL.

**Figure 2:**
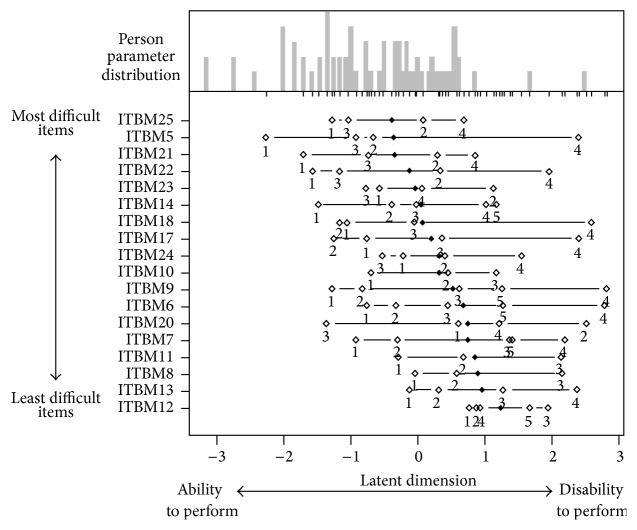
The Rasch person-item map displays the location of item (and threshold) parameters as well as distribution of person parameters along latent dimension. Difficult to easier items to perform are arranged from the top to bottom of *y*-axis; less to higher visual ability is arranged on either side of midpoint on *x*-axis. Subjects with the least visual ability (at the left side of *x*-axis) have difficulty even with the easiest items; subjects with more visual ability (at the right side of *x*-axis) have no difficulty performing any of the items. This figure shows that the items such as mental health, near activities, and dependency (located at the top of the *y*-axis) required greater visual ability and were more difficult items to perform. At the other end, color vision, social functioning, and distance activities located at the bottom of the *y*-axis required less visual ability and were less difficult items to perform.

Person-item map is shown in Figure 2. Subjects with the less visual ability (at the left side of *x*-axis) had difficulty even with the easiest items; subjects with more visual ability (at the right side of *x*-axis) had no difficulty performing any of the items. The figure shows that the items located at the top of the *y*-axis such as mental health, near activities, and dependencies required greater visual ability and as such were more difficult items to perform. At the other end, color vision, social functioning, and distance activities located at the bottom of the *y*-axis required less visual ability and were less difficult items to perform.

## 4. Discussion

The comparison between two decades, 2001 and 2011, showed that the patients visited ophthalmologists earlier though there was increasing prevalence of sight threatening retinopathy (in the 2011 cohort 12 patients; 10% of all patients with DR). This early visit is partly prompted by increasing advocacy and general health awareness and partly due to accessible health care facilities in the vicinity over a decade. Because the patients reported early more often they had no manifest retinopathy or had NPDR. Hypertension was the only significant comorbidity possibly contributing to vision threatening retinopathy. Despite these facts there was overall poor knowledge of DR, particularly regarding life style modification needed to reduce risk of DR and regarding the risk factors for development of eye complications. Though 52.5% were hypertensive, there was little awareness of its association with diabetes related eye complication. Patients with DR and the patients with higher education had positive KAP. Disturbing factors included the belief that medicines could be stopped when diabetes is controlled, that one need not see an ophthalmologist in absence of any visual symptom, and the neglect of foot care and exercise. Three other studies in India have shown poor KAP in both urban and rural population [9–11]. These studies have shown that older age group (40–49 years), higher education, and middle to higher socioeconomic status had better knowledge. Studies in other developing countries with similar socioeconomic environment as India have also shown low KAP [12–14].

NEI-VFQ has been used to assess VR-QOL in a variety of eye diseases [15–20]. These studies have consistently shown that visual acuity impacts the VR-QOL especially in domains of near and distance activities and role difficulties. We found a significant association between visual acuity and quality of life. We set the vision cut-off point at 20/60 as this is the minimum required vision for near and distance activities. Similar to ours, other studies have also shown that visual acuity drives the QOL outcomes in patients with diabetic retinopathy [21–25]. A reduction of 2 ETDRS lines seems to affect domains of driving, dependency, role limitation, and mental health [23, 24]; reduction of 3 ETDRS lines additionally affects domains of near and distance vision [22]. In our study maximum affected domains were distance and near activities. We removed items of general health and ocular pain, as they did not fit the questionnaire validation by Rasch analysis.

There was overall higher VFQ in patients with NPDR than PDR, but it was statistically not significant. This could be rather related to the fact that patients with NPDR had a relatively better vision than patients with PDR. But with increasing number of people with macular edema and vision threatening retinopathy [26] the QOL can not be related strictly to the DR (NPDR/PDR) status. In fact, several earlier studies have shown that the visual acuity (significantly reduced in diabetic macular edema and vision threating retinopathy) impacts vision related QOL [27–29]. In addition to best corrected vision, advancing age and presence of other comorbidities (chiefly hypertension) were related to poor QOL in our study. The Wisconsin Epidemiologic Study of Diabetic Retinopathy (WESDR) is another study that has looked into the impact of clinical and demographic factors such as age, comorbidity, and smoking. The WESDR study has shown that several other factors, in addition to the visual acuity, impact the VR-QOL [30].

Limitations of this study include the following: (1) the cohort comprising patients with self-reported diabetics at a tertiary eye care center may not accurately represent the general population; (2) small sample size and male preponderance may have affected the overall results; (3) the KAP study done in an eye center and in the month of World Diabetes Day (November) might have influenced the knowledge of eye involvement in DM; (4) the KAP questions designed by us were not validated in the same community; (5) the VR-QOL has considered BCVA only as a measure of vision and other clinical measures such as contrast sensitivity, central visual field, and stereopsis were not considered [6] and increase in accessible health care facility over a decade is not recorded and accounted.

At the same time the strengths of the study include (1) standardized measurement of vision using ETDRS chart, (2) clinical diagnosis of retinopathy by fellowship trained retina specialist, (3) accurate measurement of macular edema and macular status with the OCT and FFA, and (4) inclusion of a broader range of questions in KAP questionnaire in both DM and DR.

In conclusion this study showed that there is an increase in vision threating retinopathy and more number of people have hypertension, that better knowledge-attitude-practice prevails in people with higher education, and that quality of life is related to best corrected visual acuity, age, and comorbidity. We feel the current advocacy and the information-education-communication (IEC) are inadequate for growing spread of diabetes and related complications in India.

## Figures and Tables

**Table 1:** Comparison of clinical presentation in years 2001 and 2011.

	2001 DM: *n* = 472 DR: *n* = 332 Rx: 202	2011 DM: *n* = 240 DR: *n* = 118 Rx: 86	Difference in percentage between both groups	Comparison of 2 proportions (chi square test)
Any DR	332 (70.34%)	118 (49.17%)	21.17%	0.0001^*∗*^
No DR	140 (29.68%)	122 (50.83%)	21.17%	0.008^*∗*^

NPDR	192 (58%)	87% (73.7%)	15.7%	0.017^*∗*^
PDR	140 (42%)	31 (26.3%)	15.7%	0.15

≥20/60	237 (71.4%)	60 (51%)	20.4%	0.0043^*∗*^
20/400–<20/60	74 (22.2%)	46 (39%)	16.08%	0.076
<20/400	21 (6.33%)	12 (10%)	3.67%	0.75

Laser (& VEGF)	167 (82.7%)	77 (89.5%)	6.8%	0.23
Vitreous surgery	35 (17.3%)	9 (10.5%)	6.8%	0.9

Hypertension	97 (20.5%)	126 (52.5%)	32%	<0.001^*∗*^
Cardiac	17 (3.6%)	18 (7.5%)	3.9%	0.815

^*∗*^Statistically significant; DM: diabetes mellitus; DR: diabetic retinopathy; Rx: treatment; VEGF: vascular endothelial growth factor.

**Table 2:** Patient response and subgroup analysis of knowledge questions.

Question	Response	DM duration			Education			DR status		
	*n* (%)	<20 y	≥20 y	*p*	<grad	≥grad	*p*	Yes	No	*p*
	232 (100)	203 (87.5)	29 (12.5)		143 (61.6)	89 (38.4)		113 (48.7)	119 (51.3)	
(1) DM is a condition where body sugar level is high	194 (83.6)	171 (84.2)	23 (79)	0.68	118 (82.5)	76 (85.39)	0.69	95 (84)	99 (83.1)	0.99

(2) DM control requires medicine & life style changes	192 (82.8)	166 (81.7)	26 (89.6)	0.43	110 (76.9)	82 (92.13)	0.00	93 (82.3)	99 (83.19)	0.99

(3) Required life style changes in DM patient are: weight reduction/stop smoking/stop alcohol/careful diet	157 (67.7)	136 (66.6)	21 (72.4)	0.7	92 (64.3)	65 (73.03)	0.21	76 (67.2)	81 (68.0)	1

(4) Most accurate method of DM monitoring is by checking blood sugar level	178 (76.7)	156 (76.8)	22 (75.8)	0.1	108 (75.5)	70 (78.6)	0.69	88 (77.8)	90 (75.6)	0.80

(5) DM complication(s) is/are: eye, kidney, heart, foot	177 (76.3)	157 (77.33)	20 (68.9)	0.44	101 (70.6)	76 (85.3)	0.01^*∗*^	83 (73.4)	94 (78.9)	0.40

(6) DR is a complication affecting the eye	152 (65.5)	130 (64)	22 (75)	0.29	84 (58.7)	68 (76.8)	0.00	74 (65.4)	78 (65.5)	1

(7) DR affect the following eye parts: lens, retina, blood vessels	59 (25.4)	49 (24.13)	10 (34.4)	0.33	32 (22.3)	27 (30.3)	0.23	28 (24.7)	31 (26.0)	0.94

(8) Risk of eye complication is more with: longer duration of DM/poor blood sugar control/increased blood pressure	86 (37.1)	73 (35.9)	13 (44.8)	0.47	46 (32.16)	40 (44.9)	0.06^*∗*^	41 (36.2)	45 (37.8)	0.91

(9) Treatment options of eye complications of DM are: laser/medical therapy/surgery	69 (29.7)	57 (28.07)	12 (41.37)	0.21	39 (27.2)	30 (33.7)	0.37	31 (27.4)	38 (31.9)	0.54

(10) Detected on time, vision loss due to DR is treatable.	141 (61.2)	120 (59.11)	21 (72.4)	0.24	83 (58.64)	58 (65.1)	0.34	69 (61.0)	72 (60.5)	1

^*∗*^Statistically significant or tend to be significant.

**Table 3 tab3:** Patient response and subgroup analysis of attitude questions.

Question	Response	DM duration			Education			DR status		
	*n* (%)	<20 y	≥20 y	*p*	<grad	≥grad	*p*	Yes	No	*p*
	232 (100)	203 (87.5)	29 (12.5)		143 (61.6)	89 (38.4)		113 (48.7)	119 (51.3)	
(11) Upon DM control, medicines can be stopped. *Disagree*	151 (65.1)	133 (65)	18 (62.0)	0.8	88 (61.5)	63 (70.7)	0.19	72 (63.7)	79 (66.3)	0.7

(12) A DM patient should check eyes regularly. *Agree*	206 (88.8)	181 (89.1)	25 (86.2)	0.8	125 (87.4)	81 (91.0)	0.52	102 (90.2)	104 (87.3)	0.6

(13) Eyes are not affected if vision is good; examination not required. *Disagree*	154 (57.3)	133 (65.5)	21 (72.4)	0.59	84 (58.7)	70 (78.6)	**0.00**	72 (63.7)	84 (70.5)	0.32

(14) Eye complication will not occur if blood sugar is controlled. *Disagree*	99 (42.7)	85 (41.8)	14 (48.2)	0.65	50 (34.9)	49 (55.0)	**0.004** ^**∗**^	48 (42.4)	51 (42.8)	1

(15) Eye checkup can be when vision decreases since treatment can be done at any time. *Disagree.*	98 (42.2)	83 (40.8)	15 (51.7)	0.3	55 (38.4)	43 (48.3)	0.18	48 (42.4)	50 (42.0)	1

(16) If my eye checkup is normal, I need not show them again. *Disagree*	172 (74.1)	152 (74.8)	20 (68.9)	0.6	101 (70.6)	71 (79.7)	0.16	83 (73.4)	89 (74.7)	0.93

(17) Once my eyes are lasered, they are protected and require no further treatment. *Disagree*	108 (46.6)	93 (45.8)	15 (51.7)	0.6	61 (42.6)	47 (52.8)	0.17	62 (54.8)	46 (38.6)	**0.01** ^**∗**^

(18) I will go eye checkup only on advice of my physician. *Disagree*	100 (43.1)	89 (43.8)	11 (37.9)	0.68	61 (42.6)	39 (43.8)	0.9	51 (45.1)	49 (41.1)	0.63

^**∗**^Statistically significant.

**Table 4:** Patient response and subgroup analysis of practice questions.

Question	Response	DM duration			Education			DR status		
	*n* (%)	<20 y	≥20 y	*p*	<grad	≥grad	*p*	Yes	No	*p*
	232 (100)	203 (87.5)	29 (12.5)		143 (61.6)	89 (38.4)		113 (48.7)	119 (51.3)	
(19) Last checked blood sugar as advised	149 (64.2)	128 (63)	21 (72.4)	0.43	91 (63.6)	58 (65.1)	0.92	70 (61.9)	79 (66.3)	0.5

(20) Last checked blood pressure as advised	184 (79.3)	160 (78.8)	24 (82.7)	0.80	111 (77.6)	73 (82.0)	0.5	92 (81.4)	92 (77.3)	0.5

(21) Last gone for eye checkup as advised	190 (81.9)	166 (81.7)	24 (82.7)	1	115 (80.4)	75 (84.2)	0.57	96 (84.9)	94 (78.9)	0.3

(22) Medicines for DM being taken regularly	210 (90.5)	183 (90.1)	27 (93.1)	0.8	127 (88.8)	83 (93.2)	0.37	109 (96.4)	101 (84.8)	0.005^*∗*^

(23) Foot care practiced regularly	129 (55.6)	110 (54.1)	19 (65.5)	0.3	72 (50.3)	57 (64.0)	0.05^*∗*^	68 (60.1)	61 (51.2)	0.21

(24) Planned and controlled diet is followed	177 (76.3)	155 (76.3)	22 (75.8)	1	105 (73.4)	72 (80.8)	0.25	86 (76.1)	91 (76.4)	1

(25) Exercise being done regularly	125 (53.9)	109 (53.6)	16 (55.1)	1	71 (49.6)	54 (60.6)	0.1	60 (53.0)	65 (54.6)	0.9

^*∗*^Statistically significant.

**Table 5:** Disease spectrum (NPDR and PDR) based VFQ score.

Item	NPDR		PDR		*t*-test
	Mean	SD	Mean	SD	*p* value
Near activities	63.97	20.80	60.00	23.86	0.52
Distance activities	70.00	21.77	64.25	21.29	0.32
Social functioning	79.63	20.20	82.08	21.34	0.66
Mental health	62.96	31.04	50.79	29.57	0.12
Role difficulties	64.51	25.81	65.87	28.86	0.85
Dependency	79.51	23.18	73.81	26.64	0.39
Color vision	90.28	17.79	88.75	24.97	0.80
Peripheral vision	79.17	22.12	75.00	25.00	0.51
VFQ score	71.04	18.42	66.25	21.66	0.39

NPDR: nonproliferative diabetic retinopathy; PDR: proliferative diabetic retinopathy; SD: standard deviation.

**Table 6:** BCVA (<20/60 and ≥20/60) based VFQ.

Item	<20/60		≥20/60		*t*-test
	Mean	SD	Mean	SD	*p* value
Near activities	43.96	16.47	69.91	18.92	<0.001^*∗*^
Distance activities	47.89	17.27	75.87	18.01	<0.001^*∗*^
Social functioning	60.83	17.12	87.50	16.49	<0.001^*∗*^
Mental health	41.25	27.10	66.21	29.72	<0.001^*∗*^
Role difficulties	45.00	29.04	72.12	21.59	<0.001^*∗*^
Dependency	63.12	28.09	83.30	20.27	0.007^*∗*^
Color vision	78.75	26.00	93.98	15.31	0.021^*∗*^
Peripheral vision	63.75	23.61	83.18	20.44	0.003^*∗*^
VFQ score	51.90	16.63	76.19	15.98	<0.001^*∗*^

SD: standard deviation; ^*∗*^significant.

## Additional Points

*Main Messages*. (i) Patients with diabetes report early to eye care facility in India. (ii) Despite this early visit the presenting vision is not good. (iii) Only patients with longer duration diabetes and educated people are aware of care of diabetes.
